# Global DNA Methylation Changes in Nile Tilapia Gonads during High Temperature-Induced Masculinization

**DOI:** 10.1371/journal.pone.0158483

**Published:** 2016-08-03

**Authors:** Li-Xue Sun, Yi-Ya Wang, Yan Zhao, Hui Wang, Ning Li, Xiang Shan Ji

**Affiliations:** 1 College of Animal Science and Technology, Shandong Agricultural University, Taian, 271018, China; 2 College of Life Sciences, Shandong Agricultural University, Taian, 271018, China; The Ohio State University, UNITED STATES

## Abstract

In some fish species, high or low temperature can switch the sex determination mechanisms and induce fish sex reversal when the gonads are undifferentiated. During this high or low temperature-induced sex reversal, the expressions of many genes are altered. However, genome-wide DNA methylation changes in fish gonads after high or low temperature treatment are unclear. Herein, we compared the global DNA methylation changes in the gonads from control females (CF), control males (CM), high temperature-treated females (TF), and high temperature-induced males (IM) from the F8 family of Nile tilapia (*Oreochromis niloticus*) using methylated DNA immunoprecipitation sequencing. The DNA methylation level in CF was higher than that in CM for various chromosomes. Both females and males showed an increase in methylation levels on various chromosomes after high-temperature induction. We identified 64,438 (CF/CM), 63,437 (TF/IM), 98,675 (TF/CF), 235,270 (IM/CM) and 119,958 (IM/CF) differentially methylated regions (DMRs) in Nile tilapia gonads, representing approximately 0.70% (CF/CM), 0.69% (TF/IM), 1.07% (TF/CF), 2.56% (IM/CM), and 1.30% (IM/CF)of the length of the genome. A total of 89 and 65 genes that exhibited DMRs in their gene bodies and promoters were mapped to the Nile tilapia genome. Furthermore, more than half of the genes with DMRs in the gene body in CF/CM were also included in the IM/CM, TF/CF, TF/IM, and IM/CF groups. Additionally, many important pathways, including neuroactive ligand-receptor interaction, extracellular matrix-receptor interaction, and biosynthesis of unsaturated fatty acids were identified. This study provided an important foundation to investigate the molecular mechanism of high temperature-induced sex reversal in fish species.

## Introduction

Sex-determining mechanisms in gonochoristic fish can broadly be classified as genotypic (GSD), temperature-dependent (TSD), or genotypic plus temperature effects (GSD+TE) [1,2]. For fish species with TSD, they should have a sex ratio response to temperature within the range of temperature during development in the wild and should not have sex chromosomes [1]. However, artificially high or low temperatures during critical thermosensitive periods, which is ecologically irrelevant, also results in sex ratio changes in many fish, which are defined as the GSD+TE type. For GSD + TE, a high or low temperature can override the influence of genetics and switch the sex determination mechanisms when the gonads are undifferentiated [3]. However, long-term extreme temperature treatment even resulted in the sterility in female Nile tilapia [4].

In 2011, Navarro-Martín et al. first described DNA methylation of *cyp19a* (cytochrome P450, family 19, subfamily a), which regulated the *cyp19a* mRNA expression, and temperature effects on sex ratios in European sea bass [5]. During high or low temperature-induced sex reversal in fish species, the expressions of many genes are altered, such as *cyp19a1a*, *foxl2* (forkhead box L2), *sox9* (sex-determining region Y box 9), *dmrt1* (doublesex and mab-3 related transcription factor 1), *ERα*, and *ERβ* [6–11]. Thus, a high or low temperature could cause a global change of DNA methylation and gene expression. To the best of our knowledge, studies focusing on genome-wide DNA methylation changes in the gonads after high or low temperature treatment in Nile tilapia (*Oreochromis niloticus*) have not been performed.

Nile tilapia is the third most important aquaculture fish after carp and salmon, and the males grow significantly faster than females. A considerable amount of previous research has made the sex determination mechanism Nile tilapia relatively clear [12]. In Nile tilapia, a GSD + TE sex determination fish, high temperature treatment applied after hatching (around 10 days post-fertilization) and lasting from 10 to 28 days, significantly increased the male ratio[3,13–18]. Therefore, the thermal control of sex in Nile tilapia is an economic and environmentally friendly method [19]. In 2012, the whole genome of Nile tilapia was sequenced. Thus, Nile tilapia is an appealing model to study the molecular mechanisms underlying GSD + TE.

Methylated DNA immunoprecipitation sequencing (MeDIP) is a recently devised method to determine the distribution of DNA methylation within functional regions (e.g., promoters) or in the entire genome, robustly and cost efficiently [20]. In this study, we used Nile tilapia as a model to perform a genome-wide survey of DNA methylation differences in female and male gonads between control and high temperature-induced groups using MeDIP. The objectives of present study were: 1) to identify the genomic DNA methylation changes after high temperature treatment in Nile tilapia; 2) to identify the high temperature induction-related differentially methylated regions (DMRs); and 3) to identify critical pathways associated with high temperature-induced masculinization.

## Results

### Sex identification and gonad histology

There are strong maternal and paternal effects on female-to-male sex reversal in Nile tilapia under high temperature [21]; therefore, three families were developed. For sex identification, 177 4-month-old Nile tilapia in the control group and 187 in the high temperature-induced group from F8were sacrificed, and the percentage of males in the high temperature-induced group was 97.7% (49.7% for control group). F8 had the highest male percentage; therefore, the gonads from F8 CF, CM, TF, and IM were selected for MeDIP-seq.

**Fig 1:**
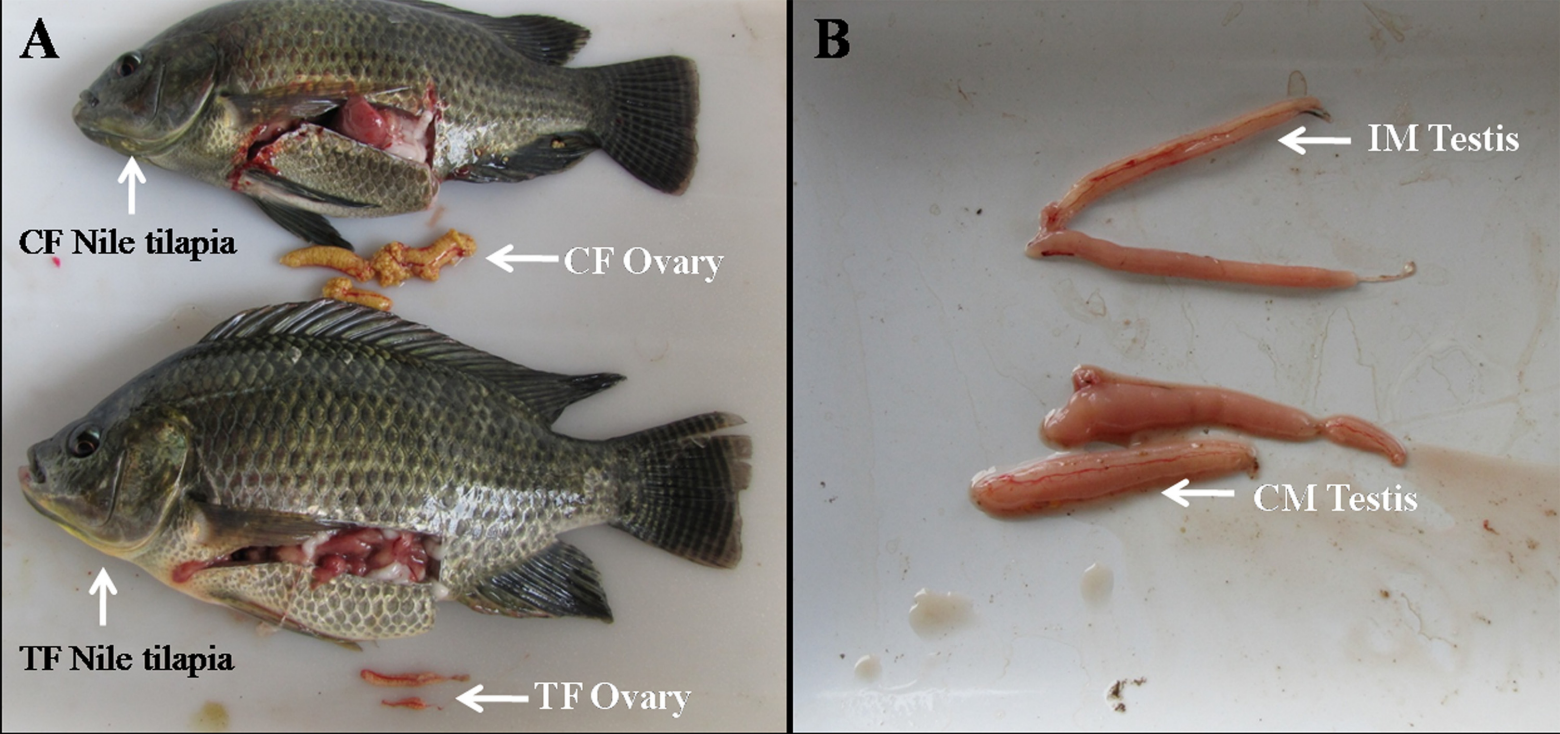
Morphology of ovary and testis of Nile tilapia exposed to control (28°C) and high temperature (36°C). **(A)** Ovaries of control and 36°C treated fish after extended rearing of high temperature treated fish in the water at normal temperature for 188 days. The ovary in TF (36°C treated females) is significantly smaller than that in CF (control females); **(B)** Testis of control(28°C) and 36°C treated fish after extended rearing of high temperature treated fish in the water at normal temperature for 188 days.

**Fig 2:**
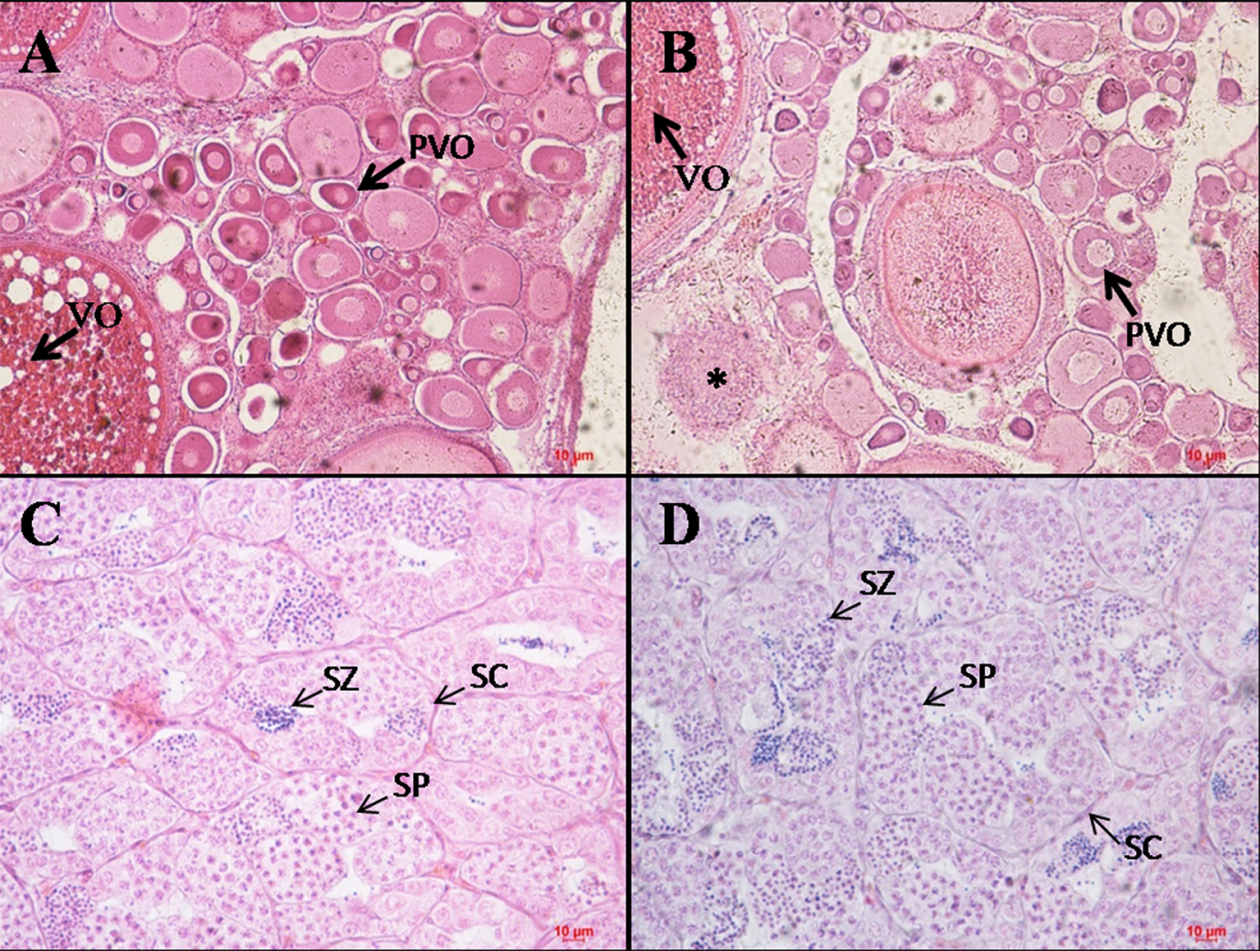
Histology of ovary and testis of Nile tilapia exposed to control (28°C) and high temperature (36°C). Histology of the control (28°C) **(A)** and high temperature (36°C) treated; **(B)** fish ovary after extended rearing of high temperature treated fish in the water at normal temperature for 188 days, with decrease of germ cells in high temperature treated fish ovary H&E×100; **(C, D)** Histology of the control (28°C); **(C)** and high temperature (36°C) treated; **(D)** fish testis after extended rearing of high temperature treated fish in the water at normal temperature for 188 days H&E×400.Scalebars represent 10 μm. PVO = oocytes at the pre-vitellogenic stage; VO = oocytes at the vitellogenic stage; SP = spermatocyte; SZ = spermatozoa; SC = Sertoli cells; *: abnormally developed oocyte.

After extended rearing of high temperature treated fish in the water at normal temperature for 188 days, we found that both females and males of high temperature treated and control fish could be sexually matured. The 36°C high temperature treated fish had smaller ovary than control fish (Fig 1). Although there are also oocytes at the pre-vitellogenic and vitellogenic stage in the ovary of 36°C high temperature treated fish compared with control, a decrease of germ cells in high temperature treated fish ovary and some abnormally developed oocytes were observed (Fig 2). The testis of control and 36°C high temperature treated fish was not significantly different in morphology (Fig 1). Histological analysis showed there were also not significant differences in the number of spermatocyte and spermatozoa (Fig 2).

### Summary of MeDIP data

Approximately 16.15 Gb of data were produced from four gonad samples, among which 85.88% were clean reads (approximately 13.75 Gb). Then, 12.39 Gb of non-duplicate reads (approximately 90.12% of the clean reads) were obtained after removing the reads showing the same mapping locations in each sample, which were regarded as potentially duplicated clones generated via PCR amplification. Last, 6.54 Gb of non-duplicate reads (approximately 47.56% of the clean reads) were uniquely aligned to Nile tilapia genome Orenil1.1 (Table 1). CpG sites with a read depth (reads per kilobase of transcript per million mapped reads, RPKM) of more than 10 were identified as high-confidence CpG sites. The CpG sites meeting this threshold were 76.6% (CF), 56.2% (CM), 68.3% (TF), and 73.1% (IM) (S1 File).

**Table 1:** Summary of MeDIP-Seq data.

Sample	Number of raw data	Raw reads (Gb)	Number of clean data	Cleans reads (Gb)	Clean data ratio (%)	Non-duplicate ratio (%)	Unique mapped ratio (%)
Control-F	128,055,474	6.40	105,101,871	5.25	82.08	86.07	47.49
Control-M	50,323,361	2.52	47,468,675	2.37	92.33	95.87	47.36
Treated-F	69,696,162	3.48	55,024,475	2.75	78.95	93.25	47.43
Induced-M	75,132,883	3.75	67,759,606	3.38	90.19	85.31	47.97
Total/Average	323,207,880	16.15	275,354,627	13.75	85.88	90.12	47.56

### DNA methylation changes on a genome-wide scale

**Fig 3:**
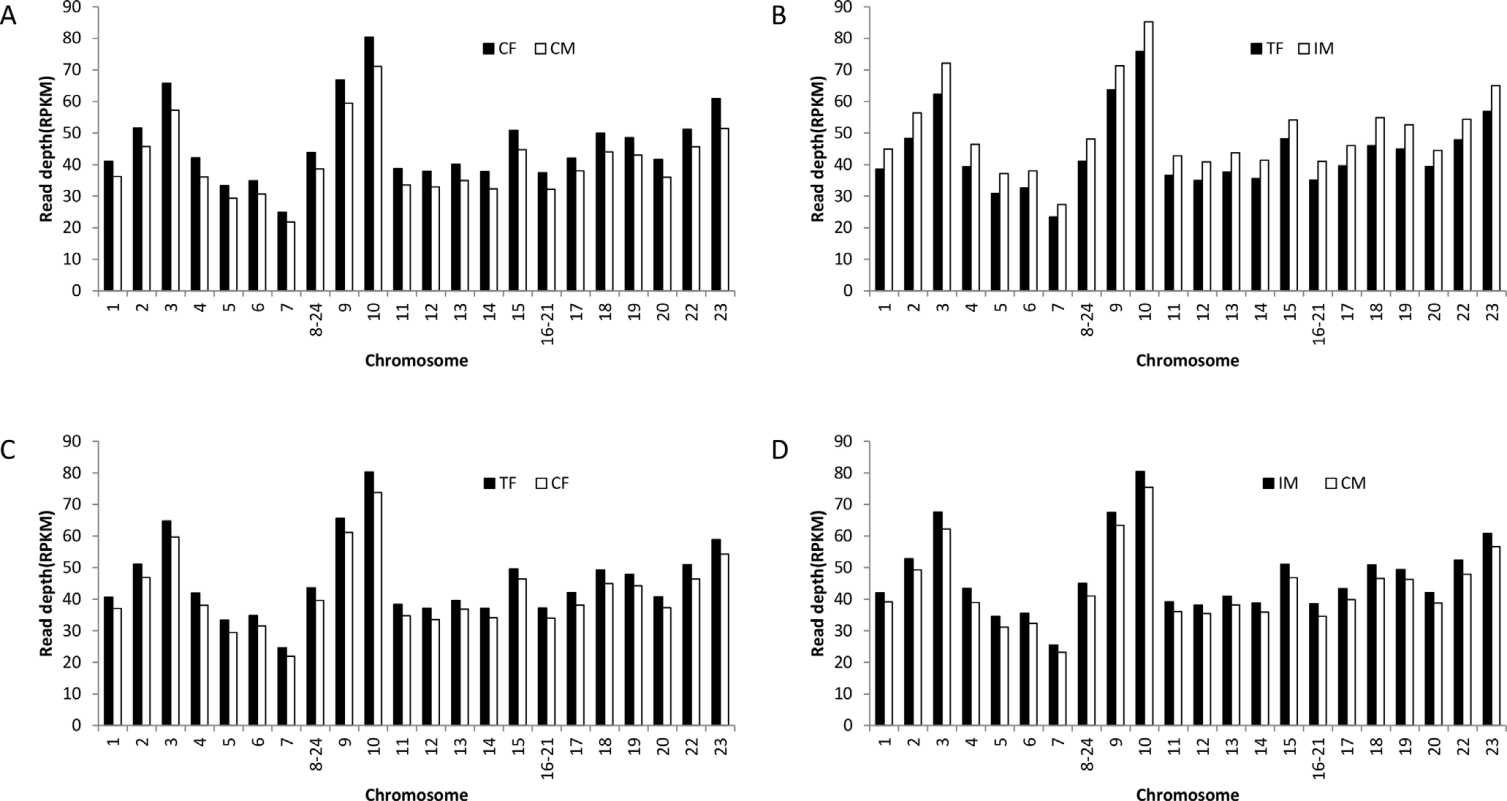
**Chromosomal profiles of DNA methylation among (A)** CF/CM**; (B)** TF/IM**; (C)** TF/CF**; (D)** IM/CM. CF: control females. CM: control males. TF: high temperature-treated females. IM: high temperature-induced males.

To study DNA methylation changes on a genome-wide scale, we investigated the chromosomal profiles of DNA methylation among CF/CM, TF/IM, TF/CF, and IM/CM. The DNA methylation level in CF was higher than that in CM for various chromosomes (Fig 3A). High temperature-induced methylation alterations in the chromosomal landscape were observed. Both Nile tilapia females and males showed an increase in methylation levels on various chromosomes after high-temperature induction (Fig 3C and 3D). Furthermore, there was a higher DNA methylation level in IM compared with TF (Fig 3B), which was different from the result between CF and CM. Thus, high temperature treatment had a greater effect on the DNA methylation level of males compared with females.

**Fig 4:**
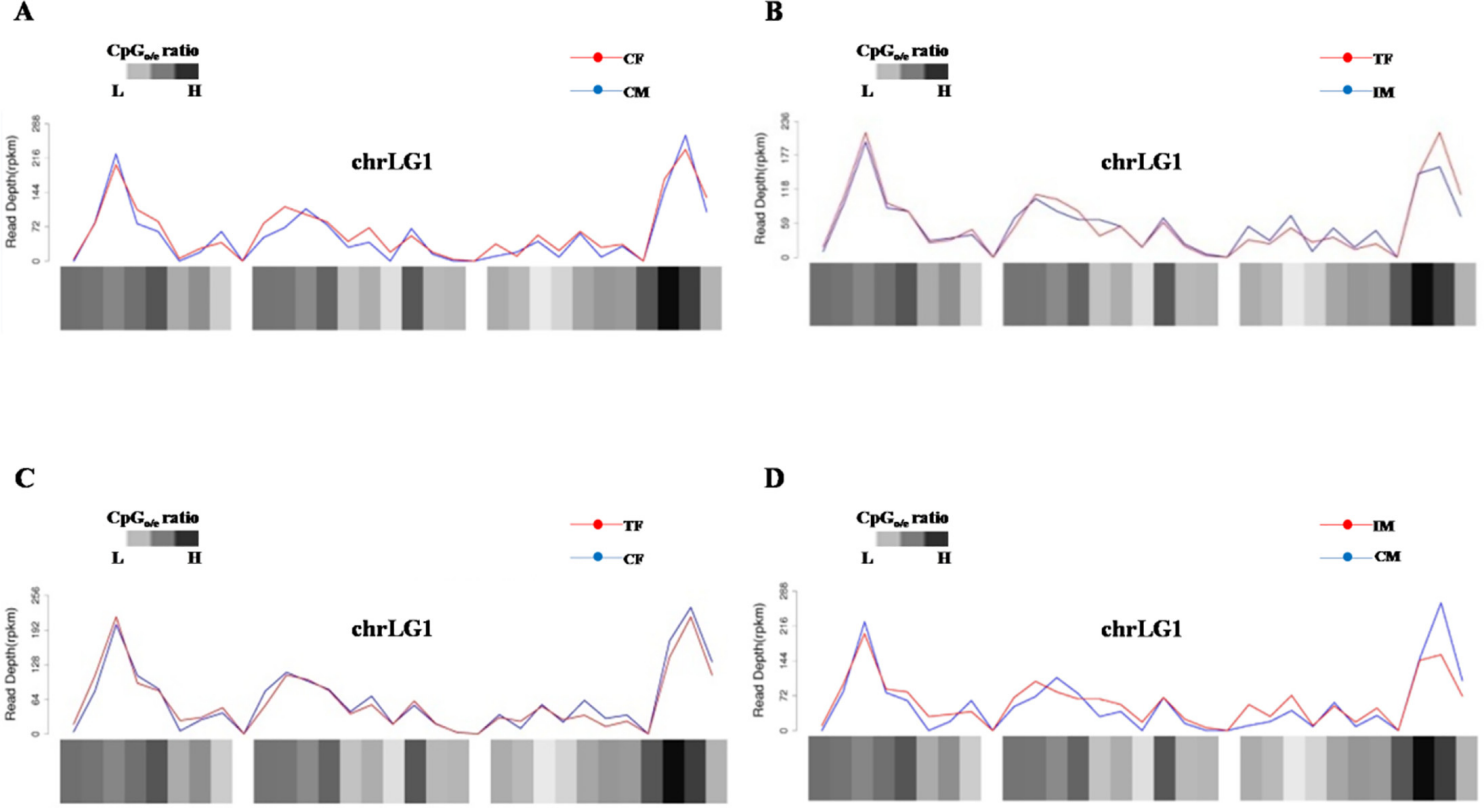
Distribution of DNA methylation on Nile tilapia chromosome 1. **(A)** CF/CM; **(B)** TF/IM; **(C)** TF/CF; **(D)** IM/CM. CF: control females. CM: control males. TF: high temperature-treated females. IM: high temperature-induced males.

A visual inspection of the chromosomal profiles along the chromosomes among CF/CM, TF/IM, TF/CF, and IM/CM was performed. Many DMRs on each chromosome were found (S2 File). For instance, the many differential methylation regions on chromosome 1 among CF/CM, TF/IM, TF/CF, and IM/CM are depicted shown in Fig 4. Additionally, the ratio of observed to expected CpG dinucleotides (CpGo/e) was used to predict the methylation status. The chromosomal profiles indicated that regions with high methylation levels tended to have high CpGo/e ratios (S2 File). The DNA methylation changes on a genome-wide scale after high-temperature induction indicated that DNA methylation acts as an important factor that connects environmental temperature and sex reversal in Nile tilapia.

### DMRs

We identified 64,438(CF/CM), 63,437(TF/IM), 98,675 (TF/CF), 235,270 (IM/CM), and 119,958 (IM/CF) DMRs in Nile tilapia gonads (S1, S2, S3, S4 and S5 Tables), representing approximately 0.70%(CF/CM), 0.69%(TF/IM), 1.07% (TF/CF), 2.56% (IM/CM), and 1.30% (IM/CF) of the length of the genome (Table 2). The DMRs were mostly located in intergenic regions of the chromosomes. The number of DMRs located in the gene bodies and promoters were 229 (CF/CM), 204 (TF/IM), 472 (TF/CF), 1,100 (IM/CM), and 541 (IM/CF) (Table 2).

**Table 2:** Summary of differentially methylated regions (DMRs).

DMR type	Number of DMRs	Percentage of genomic length*	Number of DMRs locating in the gene bodies and promoters
CF/CM	64438	0.70	229
IF/IM	63437	0.69	204
IF/CF	98675	1.07	472
IM/CM	235270	2.56	1100
IM/CF	119,958	1.30	541

*Total length of all DMRs relative to the length (approximately 0.92 billion bp) of the Nile tilapia genome (Orenil 1.1).

To further explore potential distribution biases in the DMRs, we analyzed the percentage of the DMRs in various genomic regions. We defined 10 categories of functional genomic elements, including promoter, exon, intron, intergenic, SINE, LINE, simple repeat, repetitive sequence, low complexity, and others. The distribution pattern of DMRs in six functional genomic elements was almost identical among CF/CM, TF/IM, TF/CF, IM/CM, and IM/CF (S1 Fig). A large number of DMRs were located at intergenic regions and only a small proportion of DMRs overlapping with SINEs was observed, supporting previous findings that the majority of methylated CpGs are located in intergenic regions [22].

**Fig 5:**
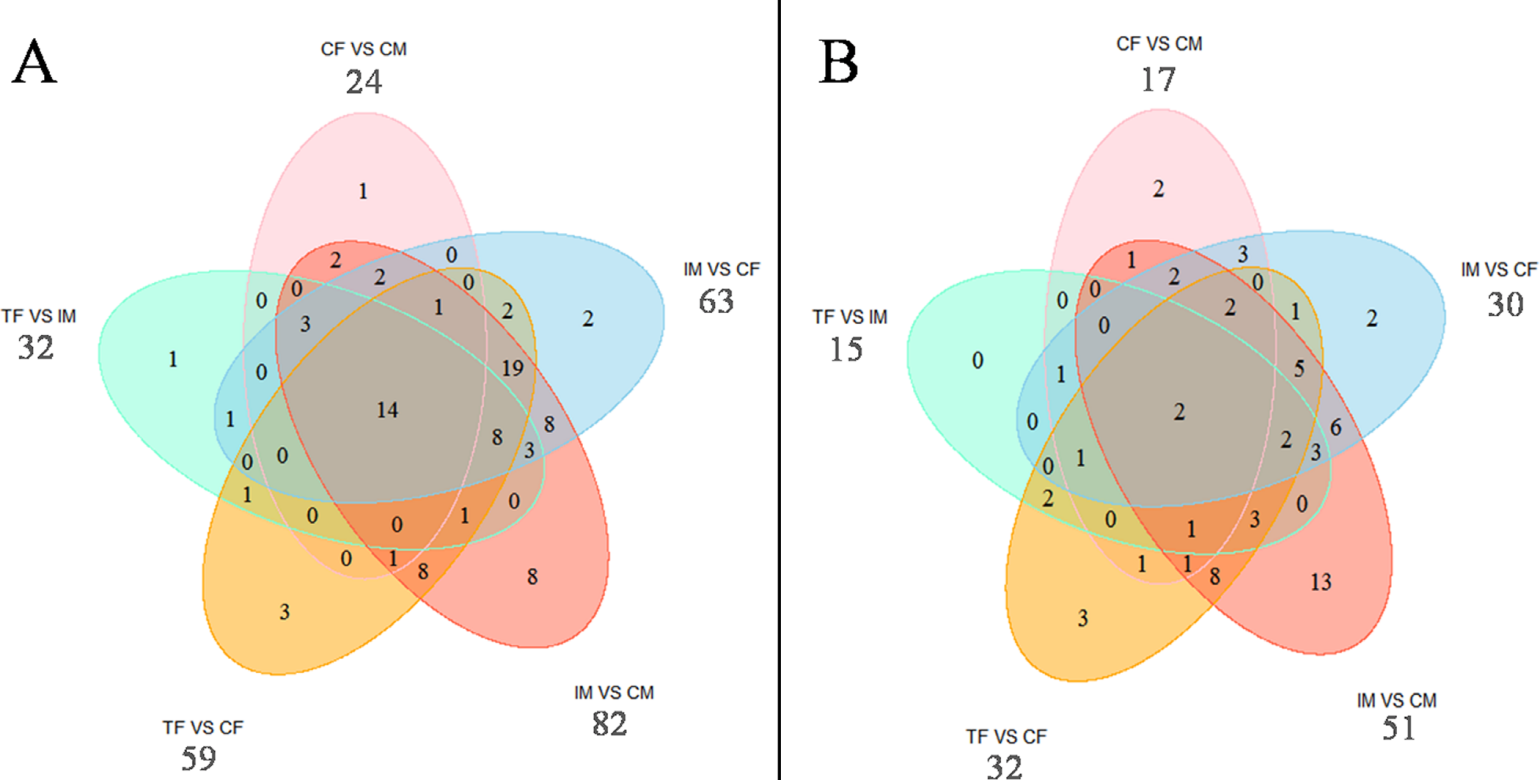
**Venn diagram of the numbers of genes with DMRs in the gene body (A) and in the promoter (B)**. CF: control females. CM: control males. TF: high temperature-treated females. IM: high temperature-induced males.

As shown in the Venn diagram in Fig 5, 89 and 65 genes that exhibited DMRs in their gene bodies and promoters, respectively, were mapped to the Nile tilapia genome, respectively. Among 24 genes with DMRs in their gene bodies in the CF/CM, 23 were included in the list of genes with DMRs in their gene bodies in the IM/CM, and 17, 16 and 20 in the TF/IM, TF/CF, and IM/CF, respectively (Fig 5A). Fifty-nine genes with no DMRs in the CF/CM showed DMRs in their gene bodies in the IM/CM. Similarly, 43 and 42 genes showed DMRs in their gene bodies in the TF/CF and IM/CF. Among 17 genes with DMRs in their promoters in the CF/CM, an intersection of the CF/CM and TF/CF revealed eight and eleven common genes in CF/CM and IM/CF, respectively (Fig 5B).

### Biological pathways associated with high-temperature induction

KEGG pathway annotations were performed using the KEGG Automatic Annotation Server with the bidirectional best hit information method [23]. Pathways with a P value <0.05 were significantly enriched and are listed in S6 Table. The identified important pathways include neuroactive ligand-receptor interaction, extracellular matrix-receptor interaction, biosynthesis of unsaturated fatty acids, ribosome, natural killer cell-mediated cytotoxicity, and alpha-linolenic acid metabolism. Eighteen genes in CF/CM and IM/CM and 16 genes among TF/IM, TF/CF, and IM/CF were identified as involved in the neuroactive ligand-receptor interaction pathway. In the neuroactive ligand-receptor interaction pathway, some receptor genes, such as *GnRH receptor type 2* (gonadotropin releasing hormone type 2), *gth-rII* (gonadotropin receptor II), and *gpr54* (G-protein coupled receptor), were included in IM/CM, TF/CF, or TF/IM. In the biosynthesis of unsaturated fatty acids pathway, *hsd17b12* (17β- hydroxysteroid dehydrogenase 12) and *acyl-CoA oxidase 1* were included in IM/CM, IF/CF, or IF/IM.

### Identified genes involved in high temperature-induced masculinization

A potential role of DNA methylation in regulating gene expression has been proposed [22,24–26]. *nr0b1a* is one of the major factors implicated in tilapia sex differentiation through transcriptional regulation of aromatase gene and estrogen production [27]. In this study, we found *nr0b1a* (Orphan nuclear receptor DAX1) with higher methylation status (RPKM = 80.41) in IM compared with CF (RPKM = 35) (S5 Table). *er-b2* coupled with estrogen acts on *cyp19a1a* promoter and repressed *cyp19a1a* expression [27]. Our MeDIP result indicated that lower methylation status of *er-b2* (RPKM = 1.67) in IM compared with CF (RPKM = 13) (S5 Table). *gsdf* was found to act as male sex initiator [28] and we observed it with lower methylation status in IM (RPKM = 7.7) compared with CM (RPKM = 35.0) (S4 Table).

**Fig 6:**
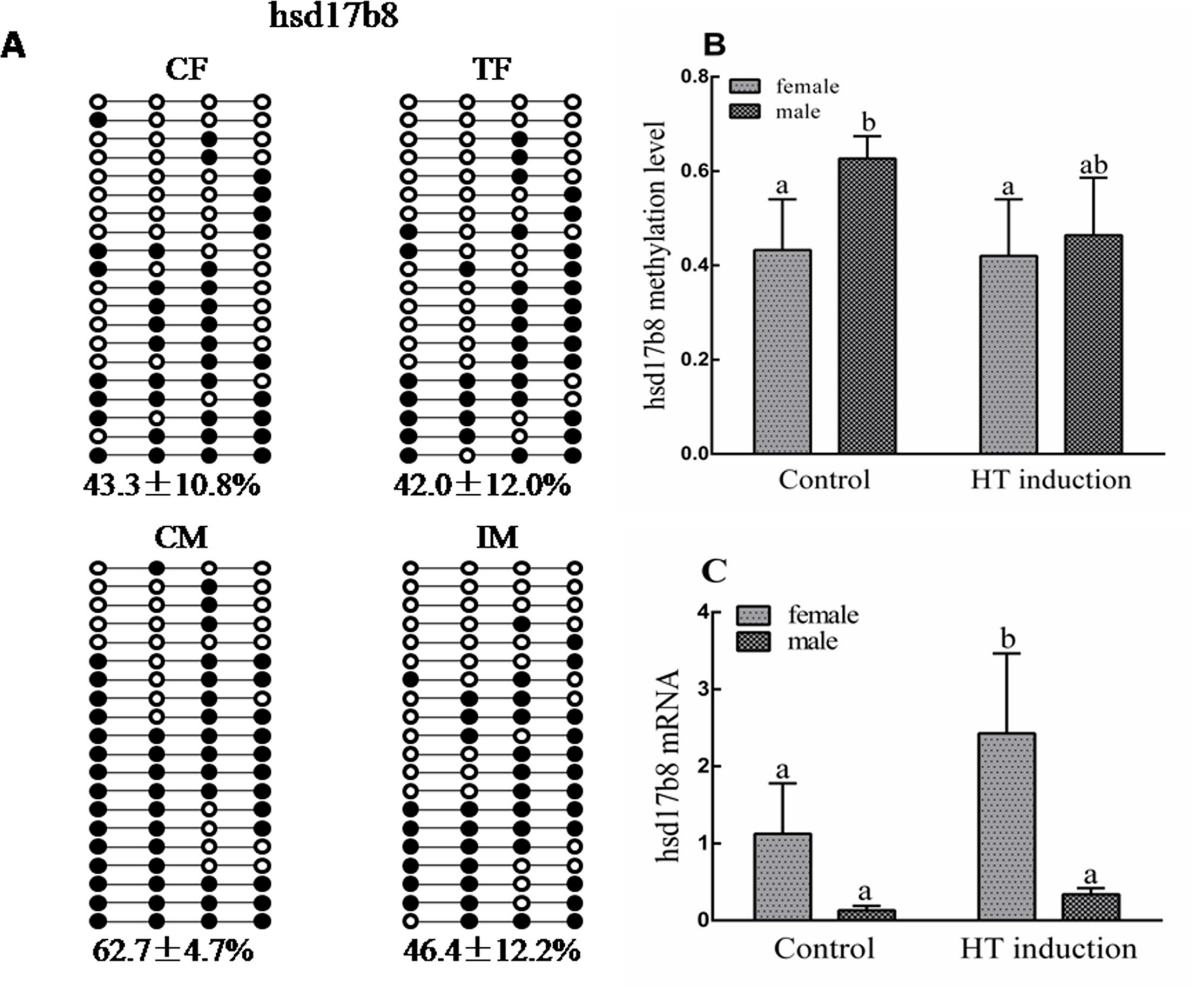
Differential DNA methylation in the *hsd17b8* promoter. **(A)** Validation of CpG methylation by bisulfite sequencing. Ten subclones were selected for BSP analysis. The solid circles represent the methylated CpG sites, and the open circles represent the unmethylated CpG sites; **(B)** Methylation levels of four groups; **(C)** Comparison of gene expression levels among four groups (CF, CM, TF, IM). CF: control females. CM: control males. TF: high temperature-treated females. IM: high temperature-induced males.

**Fig 7:**
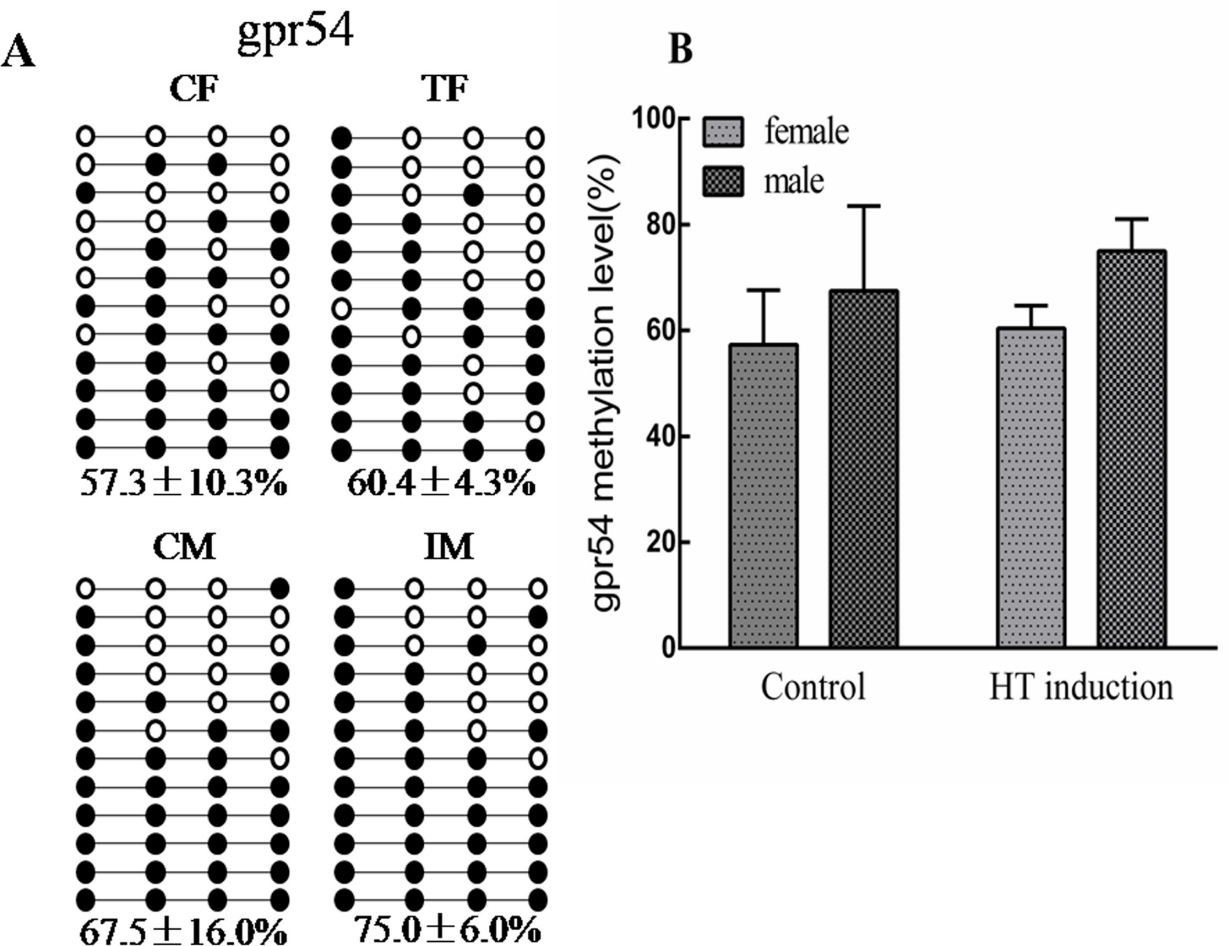
Differential DNA methylation in *gpr54* gene body validated by BSP. **(A)** Ten subclones were selected for BSP analysis. The solid circles represent the methylated CpG sites, and the open circles represent the unmethylated CpG sites; **(B)** Methylation levels among four groups (CF, CM, TF, IM). CF: control females. CM: control males. TF: high temperature-treated females. IM: high temperature-induced males.

To further highlight the potential roles of genes involved in high temperature-induced masculinization, several genes with DMRs in their gene bodies and promoters were confirmed using bisulfite sequencing PCR (BSP) or quantitative real-time PCR (RT-qPCR). For example, HSD17Bs are documented to be enzymes for sex steroid hormone synthesis, reversibly interconverting 17-hydroxy and 17-keto steroids [29–31]. The MeDIP result indicated lower methylation status (RPKM = 0) of *hsd17b8* in IM compared with CM (RPKM = 22). The BSP result showed a lower methylation status of this gene in both TF and IM compared with CF and CM. Correspondingly, RT-qPCR verified that its mRNA expression was up-regulated in both IF and IM compared with CF and CM (Fig 6). *gpr54* is the receptor of kisspeptin and can stimulate the release of gonadotropin [32,33]. The hypermethylation of its gene body was observed in both sexes of the high temperature induction group using BSP (Fig 7).

## Discussion

### Global DNA methylation changes after high-temperature induction

This study provided a comprehensive analysis of genome-wide DNA methylation patterns in the gonads of high temperature-induced Nile tilapia. To the best of our knowledge, this is the first study to explore the global methylation profile of XX and XY gonads after high-temperature induction. First, we found that the DNA methylation level in CF was higher than that in CM on various chromosomes. In half-smooth tongue sole, the overall methylation levels were consistently enhanced by 10% in testes compared with ovaries, except for the W chromosome [34]. The difference in global methylation profile of the gonads between females and males indicated the important role of epigenetic modification in fish gonad development. Second, we found that Nile tilapia females and males showed an increase in methylation levels on various chromosomes after high-temperature induction. A visual inspection of the chromosomal profiles along the chromosomes among CF/CM, TF/IM, TF/CF, and IM/CM were performed and many DMRs on each chromosome were found. There is increasing evidence that the environment might cause changes in organisms via epigenetic modification. Methylation of CpGs can undergo extensive changes during cellular differentiation or under various treatments [35]. For instance, transient excess androgen exposure at 26 to 56 h post-fertilization can result in transgenerational alterations in the zebrafish ovarian epigenome. However, androgen exposure at 21–28 days post fertilization did not affect zebrafish ovarian DNA methylation status [36]. Enzyme linked immunosorbent assays showed that *PGE2* could increase global DNA methylation of human fetal and adult lung fibroblasts dose dependently after 48 h of treatment [37]. These results suggested that patterns of DNA methylation are highly plastic and sensitive to environmental cues. Therefore, it is meaningful to investigate the epigenetic changes during high-temperature induced masculinization.

### DMRs

In the early differentiation of Nile tilapia gonads, high temperature can result in epigenetic changes of some genes. In the CF/CM, 24 genes and 17 genes with DMRs in their gene bodies and promoters were found, respectively. More than half of the genes with DMRs in CF/CM were also included in the IM/CM, TF/CF, and TF/IM. Previous studies have verified that the DNA methylation level of sex-biased genes plays important roles in epigenetic regulation of sex reversal mediated by temperature. For instance, the *cyp19a* promoter DNA methylation levels were twice as high in the male gonads compared with females in the 1-year-old European sea bass [4]. The high temperature induction results showed that *cyp19a* was involved in DNA methylation-mediated sex ratio shifts in the European sea bass. In alligators, incubating at a male-producing temperature (MPT) increased *cyp19a1* promoter methylation and reduced its expression levels compared with incubating at a female-producing temperature (FPT), whereas there is an opposite tendency in *sox9* [38]. However, many genes with no DMRs in the CF/CM showed DMRs in their gene bodies or promoters in the IM/CM, TF/CF, TF/IM, and IM/CF, such as nr0b1, er-b2 and gsdf. These genes could be good candidates for investigating epigenetic regulation mechanisms during high temperature-induced masculinization in Nile tilapia.

Previous studies have shown that treatment with steroids or aromatase inhibitor could directly induce the sex reversal of females in Nile tilapia [39,40], indicating the important role of sex steroids in regulating sex development. The mRNA or DNA methylation changes of*cyp19a1a* (the key enzyme of estrogen synthesis) and its transcription factors such as *foxl2* and *ER*, have been found during high temperature induced masculinization in Nile tilapia [27, 41, 42]. In this study, lower methylation status of tilapia *hsd17b8*, catalyzing the conversion of testosterone to androstenedione and the inter-conversion between estrone and estradiol [31], was found in IM compared with CM. RT-qPCR also verified that its mRNA expression was upregulated in both IF and IM compared with CF and CM. In the future, we will explore the role of*hsd17b8*during high temperature-induced masculinization in Nile tilapia.

### Neuroactive ligand-receptor interaction pathway

In this study, we identified many pathways associated with fish sex reversal under high temperature induction, providing a valuable resource for further research. Among these pathways, the neuroactive ligand-receptor interaction pathway is an attractive target because multiple receptors on the plasma membrane, which are associated with cell signaling, are located in this pathway [43,44]. In this study, we found DMRs in the gene body or promoters of neuroactive ligand-receptor interaction pathway genes *GnRH receptor type 2*, *gth-rII*, and *gpr54* in IM/CM, IF/CF, or IF/IM. *GnRH receptor type 2* and *gth-rII* mediate the role of gonadotropin-releasing hormone and gonadotropic hormone, respectively, in the hypothalamus-pituitary-gonad axis. The *kisspeptin/gpr54* signaling system stimulates gonadotropin release [45,46]. The gonadotropins act on the gonads to control sex hormone production. Furthermore, this sex steroid ratio depends on the activity of the aromatase, the product of the *cyp19a* gene, which irreversibly converts androgens into estrogens. In 2011, Navarro-Martín et al. found that exposure of undifferentiated sea bass larvae to high temperature increased *cyp19a* promoter methylation levels in both females and males, which in turn affected gene expression, estrogen synthesis, and hence the sex ratios [4]. Previous studies also showed that the expression *foxl2*, which upregulates *cyp19a1a* in tilapia [47], was suppressed in the temperature-masculinized XX female tilapia from 17 to 19dpf onwards [10,11] Concerning the possible roles of sex hormone production during high temperature-induced masculinization in Nile tilapia, our results indicated that *GnRH receptor type 2*, *gth-rII* and *gpr54*, which mediate the release of gonadotropin-releasing hormone and gonadotropin hormone, could play important roles in epigenetic regulation during high temperature-induced masculinization in Nile tilapia.

In summary, the present study showed an increase in methylation levels on various chromosomes in both sexes after high-temperature induction. We also identified many genes with DMRs in the high-temperature induction group compared with the control. We identified several pathways that were potentially involved in the connection between environmental temperature and sex ratios in species with TSD or GSD + TE. Collectively, this study provides a valuable resource to investigate the molecular mechanisms of fish sex reversal under high-temperature induction.

## Materials and Methods

### Fish culture and family development

Eighteen 3-year-old Nile tilapias, 12 females and six males, were collected from the Shandong Institute of Freshwater Fisheries (Jinan, Shandong, China), and reared in 40-m^3^ tanks in the experimental base of Shandong Agricultural University (Taian, Shandong, China). The fish were fed pelleted tilapia food of appropriate size once daily. After 2 weeks, the 18 tilapias were divided into six groups. Each group contained two females and one male cultured in a 20-m^3^ tank under a natural photoperiod and water temperature (23–29°C). The fish’s mouths were checked every 5 days. If there were embryos in the mouth, the embryos were taken out and cultivated artificially in 0.5-m^3^aquaria as a family, independently. There was one aerating stone in each 0.5-m^3^aquariumand the water temperature was controlled at 28–29°C.

### High temperature-induced masculinization

The high temperature-induced masculinization of Nile tilapia was performed as previously described [3,11,13]. Briefly, about 500 larvae at 10 days post fertilization (dpf) from each family were equally divided into two groups. One group served as the control and the larvae were cultivated at 28°C. The other was the high-temperature induced group and the larvae were cultured at 36°C for 12 days, which promotes male development. High-temperature induction was carried out in duplicate. Thereafter, the larvae of the high temperature-induced group were gradually adapted to 28°C again. The larvae from the control and high temperature-induced groups were transferred separately to a 20-m^3^ tank and cultured under a natural water temperature (21–30°C) for 98 days. The fish were fed pelleted tilapia food of the appropriate size two to three times one day. In total, three families were developed in this study.

### Sampling

The study was approved by Shandong Agricultural University Animal Care and Use Committee with approval number SDAUA-2013-002. In all cases, fish were treated in agreement with Shandong Agricultural University Animal Care and Use Committee. After extended rearing of high temperature treated fish in the water at normal temperature for 98 days, fish were sacrificed and one gonad was processed for sex identification using squash technique under an optical microscope. The shape of gonads, cell arrangement and cell size were used for the identification of sex. The other gonad was frozen in liquid nitrogen and stored at −80°C until further analysis. The male percentage of each family was determined and the family (F8) with the highest male percentage was used in this study. The gonads from F8 control females (CF), F8 control males (CM), F8 high temperature-treated females (TF), and F8 high temperature-induced males (IM) were used for DNA extraction.

### Gonadal histology

In order to investigate the histological difference of high temperature treated and control fish when their gonads were matured, the fish from CF, CM, TF and IM were sacrificed for histology examination after extended rearing of high temperature treated fish in the water at normal temperature for 188 days. The gonads were fixed in 10% neutral buffered formalin, then dehydrated in a series of ethanol concentrations, embedded in paraffin, sectioned at 5 μm, and stained with hematoxylin and eosin (HE). All slides were examined by light microscopy.

### DNA isolation and methylated DNA immunoprecipitation sequencing

DNA was isolated using the traditional phenol-chloroform method from the gonads of Nile tilapia in F8 (control and high temperature-induced group). The quality of the isolated DNA was measured using a NanoDrop spectrophotometer. The A260/A280 value should be 1.8–2.0 for each DNA sample to guarantee quality. DNA from four Nile tilapia of each group (CF, CM, TF, and IM) were pooled, and 5 μg of pooled DNA was used for MeDIP sequencing. The protocol for MeDIP-seq and detailed information on the construction of the MeDIP DNA library were provided in a previous report [48]. Each MeDIP library was subjected to paired-end sequencing using an Illumina Hiseq 2500 and the read length was 100 bp. The MeDIP-seq data have been submitted to the GEO database under the accession numberGSE72386.

### Analysis of MeDIP-seq data

The reads in the raw data that contained 5 “N”s and those in which over 50% of the sequence exhibited a low quality value (Phred score <5) were filtered out. The clean reads were then aligned to the Nile tilapia genome, version Orenil1.1, with less than one mismatch, using the Bowtie software (version 2.0). The Nile tilapia genome sequence Orenil1.0 was downloaded from the Ensembl database (http://www.ensembl.org; Orenil1.0 GCA_000188235.1). The average coverage or read depth for each CpG site was estimated from the number of sequenced DNA fragments covering that site. A normalization of read counts among samples was carried out based on the total sequenced DNA fragments for each sample.

To analyze easily the differences in the methylation level among various genomic regions, CpG islands (CGI) (regions with at least 200 bp having aGC percentage greater than 55% and an observed-to-expected CpG ratio greater than 65%), and CGI shores (regions located within 2 kb of a CGI) were classified into four classes: promoter, intragenic, 3′ transcript, or intergenic regions. The translation start site for many Nile tilapia genes is incomplete in genome sequence Orenil1.1; therefore, the region extending from −2200 to +300 bp from a gene’s translation start site was defined as the promoter. The gene structure was also divided into first exon, first intron, internal exons, internal introns, and last exon based on the Nile tilapia genome.

The DMRs (differential methylation regions) among various samples (CF/CM, TF/IM, TF/CF, IM/CM, and IM/CF) across the whole genome were identified using MEDIPS from Bioconductor (release 3.0; http://www.bioconductor.org). A list of overlapping regions of sequence alignment among various samples was created. If five or more CpGs in a region showed significant reads per kilobase of transcript per million mapped reads (RPKM, P < 0.05) and statistically significant peaks at a false discovery rate of 5% across samples, the region was considered a DMR.

### Bisulfite sequencing PCR

To track the DNA methylation status of several genes, we performed bisulfite sequencing PCR (BSP). The primers for BSP were designed using MethPrimer [49] and are provided in S7 Table. The bisulfite conversion of extracted gonad genomic DNA was demonstrated using a BisulFlash™ DNA Modification Kit (Epigentek, Farmingdale, NY). The targeted portion was amplified using two rounds of PCR using Takara Ex Taq (Hot Start Version). The PCR products were cloned into the pGM-T vector and transformed into *Escherichia coli* DH5α competent cells (Tiangen, Beijing, China). Five fish per each gene was used and 10–12 clones for each fish were randomly selected for sequencing. The average methylation levels per position were then computed.

### RT-qPCR

The RNA was respectively extracted from gonads using the Trizol reagent (Tiangen), according to the manufacturer’s instruction. The primers for each gene were designed using primer premier 5.0 and the primer sequences are shown in S8 Table. Quantitative real-time PCR (RT-qPCR) was conducted in Mx3000p™ real-time PCR system. The RT-qPCR reaction system (20 μL) comprised 10 μL of SYBR Premix Ex Taq (2×) (Takara, Shiga, Japan), 0.4 μL of each gene-specific primer (10 nmol), 2 μL of cDNA, and 0.4 μL of ROX reference dye ІІ. The PCR amplification procedure comprised initial denaturation at 95°C for 30 s; 40 cycles of 95°C for 5 s, 60°C for 30 s, 72°C for 30 s; followed by disassociation curve analysis to determine target specificity. The expression of *EF-1α*was used as the internal control [11,44]. Three replicates for each gene and for each individual were performed and the fluorescence intensities of each gene, as measured by cycle threshold (Ct) values, were compared by 2^- ΔΔCt^ method. PCR specificity was assessed by melting curve analysis.

## Supporting Information

S1 Fig Distribution pattern of DMRs in six functional genomic elements among CF/CM, TF/IM, TF/CF, IM/CM, and IM/CF. CF: control females. CM: control males. TF: high temperature-treated females. IM: high temperature-induced males.
(TIF)

S1 File Percentage of CpGs showing an average coverage that meets the read depth threshold over all samples. A: control females (CF). B: control males (CM). C: high temperature-treated females (TF). D: high temperature-induced males (IM). LINE: Long interspersed nuclear elements. SINE: Short interspersed nuclear elements
(PDF)

S2 File Distribution of DNA methylation on various chromosomes of Nile tilapia. A: CF/CM; B: TF/IM; C: TF/CF; D: IM/CM.CF: control females. CM: control males. TF: high temperature-treated females. IM: high temperature-induced males.
(PDF)

S1 Table List of DMRs between control females (CF) and control males (CM). (XLSX)

S2 Table List of DMRs between treated females (TF) and control females (IM). (XLSX)

S3 Table List of DMRs between treated females (TF) and control females (CF). (XLSX)

S4 Table List of DMRs between induced males (IM) and induced males (CM). (XLSX)

S5 Table List of DMRs between induced males (IM) and control females (CF). (XLSX)

S6 Table Over-represented functional gene categories for DMRs. (XLSX)

S7 Table Primers for BSP. (DOCX)

S8 Table Primers for RT-qPCR. (DOCX)
