# Supplementary figures and images for: Global DNA Methylation Changes in Nile Tilapia Gonads during High Temperature-Induced Masculinization
Source: PLoS One. 2016 Aug 3;11(8):e0158483. doi: 10.1371/journal.pone.0158483 (PMC4972363; doi:10.1371/journal.pone.0158483)

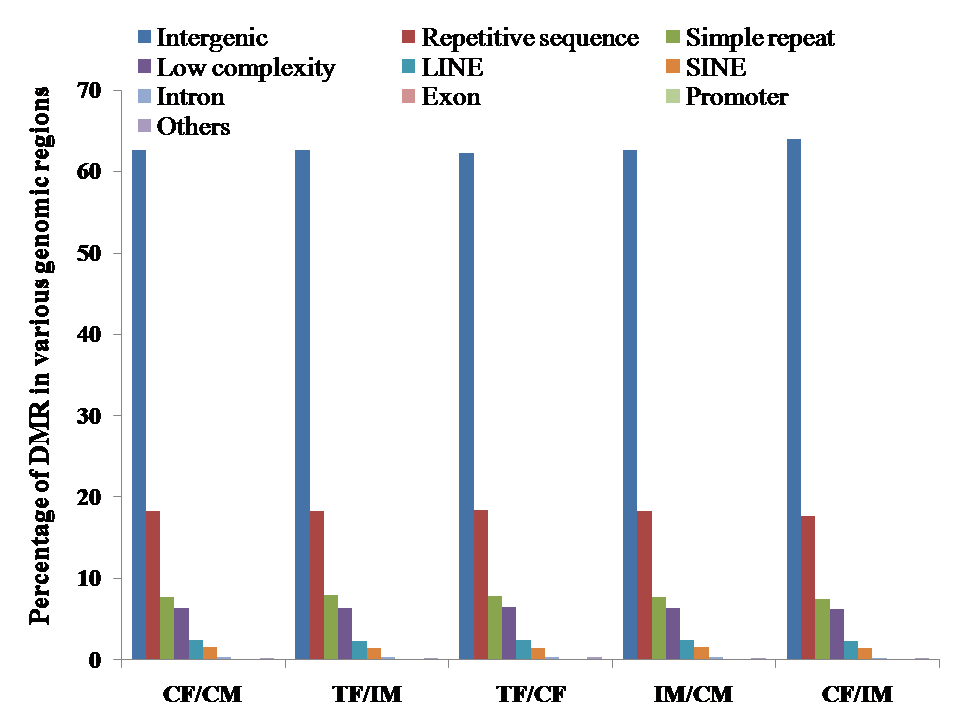

Supplement: S1 Fig — CF: control females. CM: control males. TF: high temperature-treated females. IM: high temperature-induced males. (TIF) [file pone.0158483.s001.tif]

**A**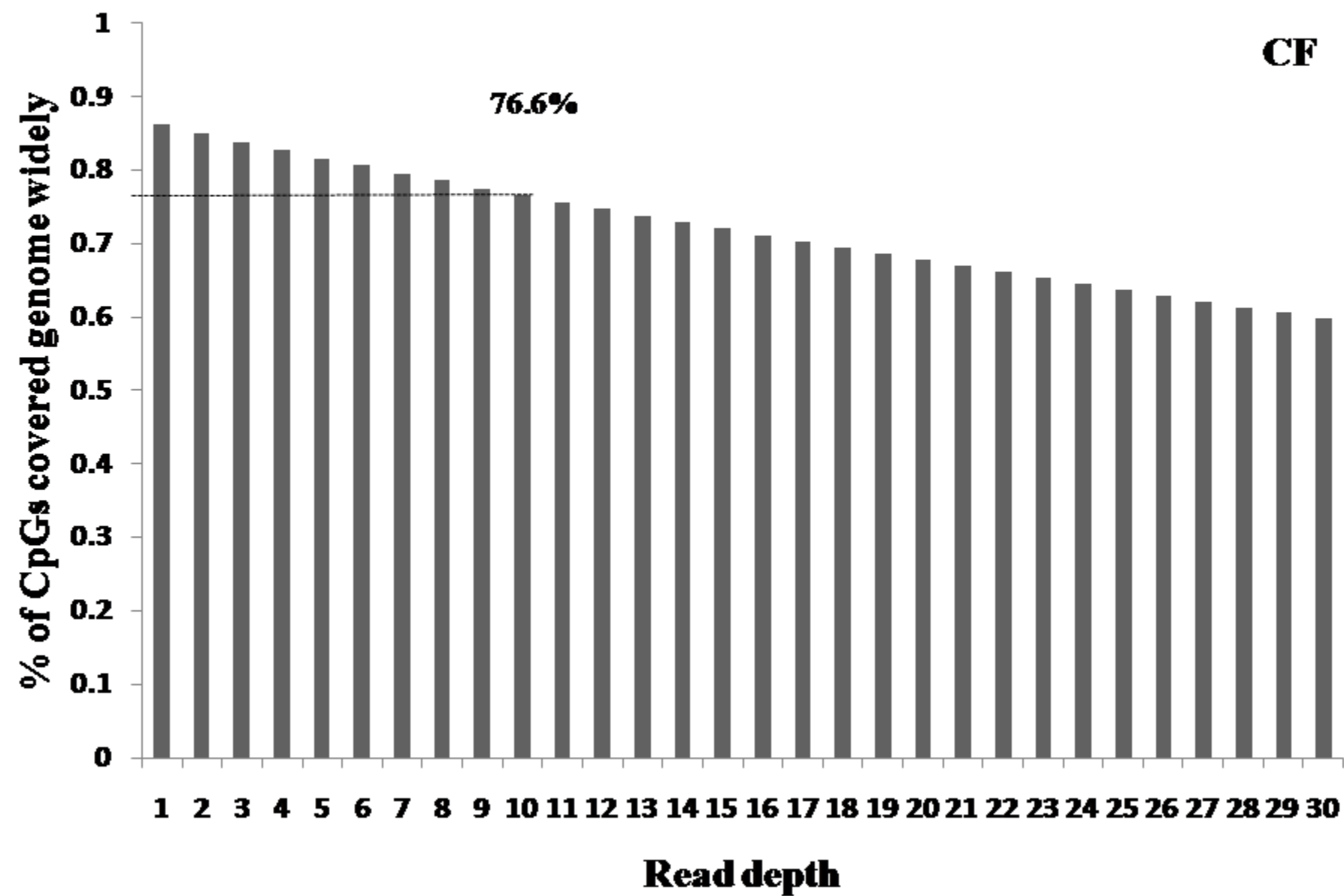

**B**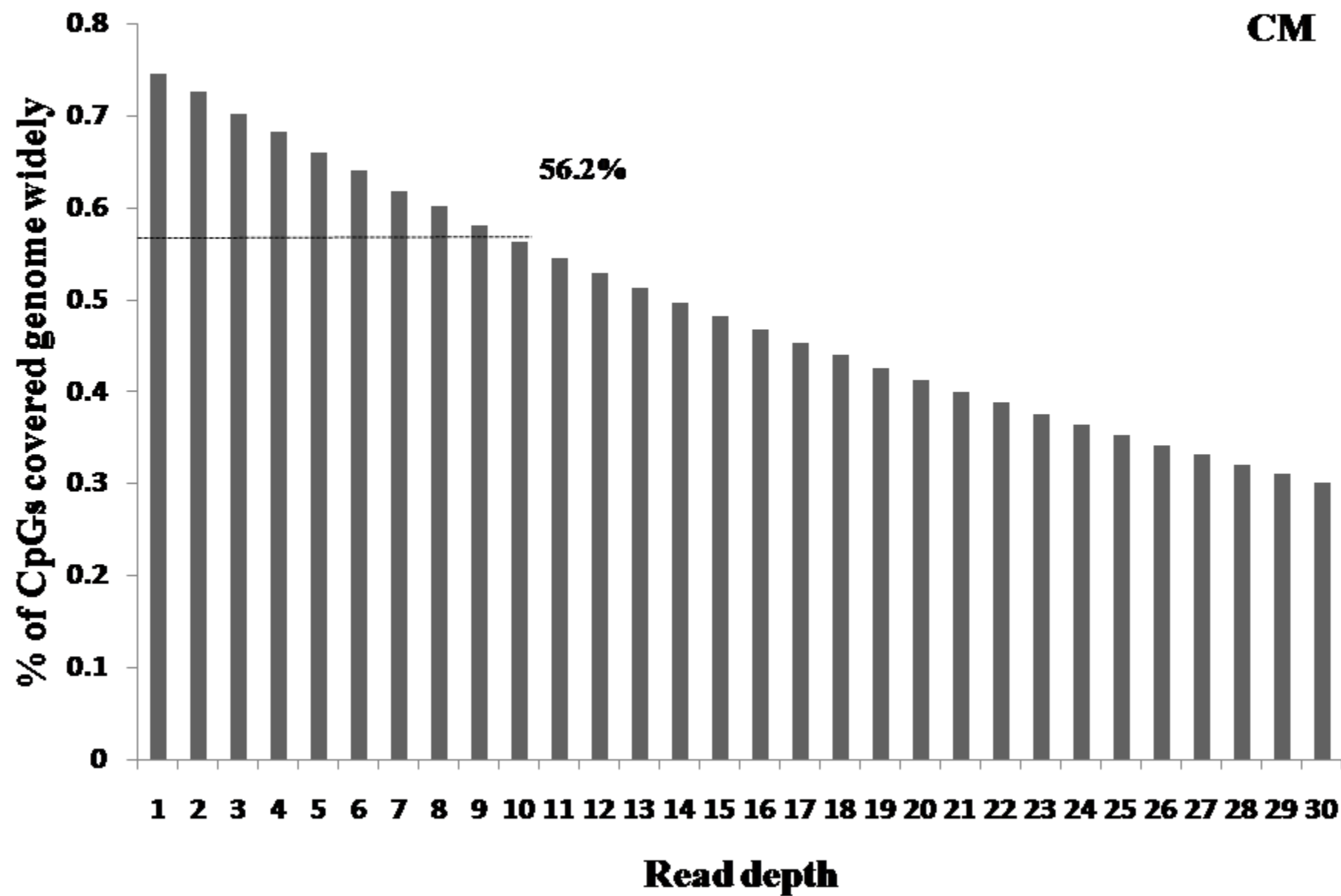

**C**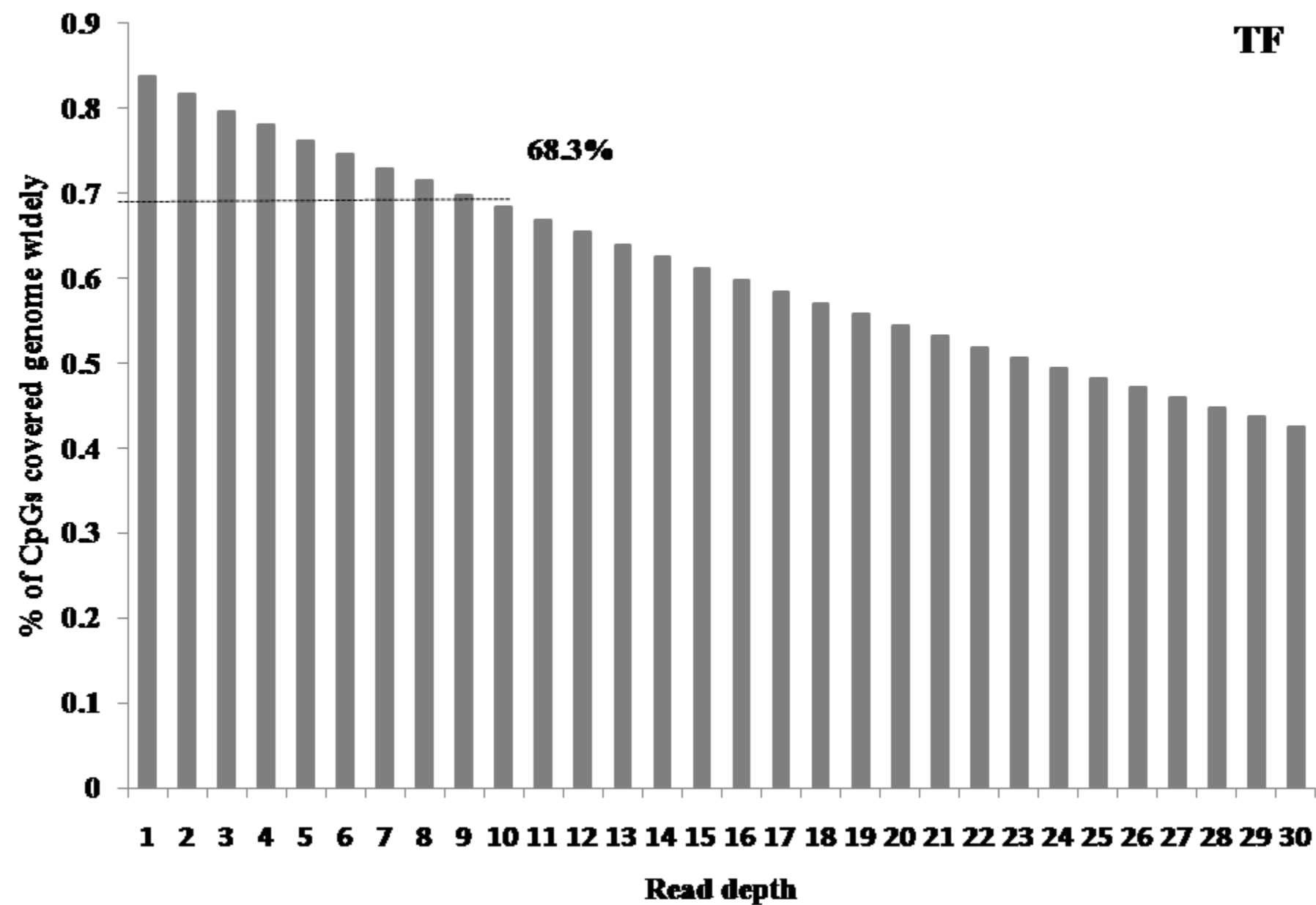

**D**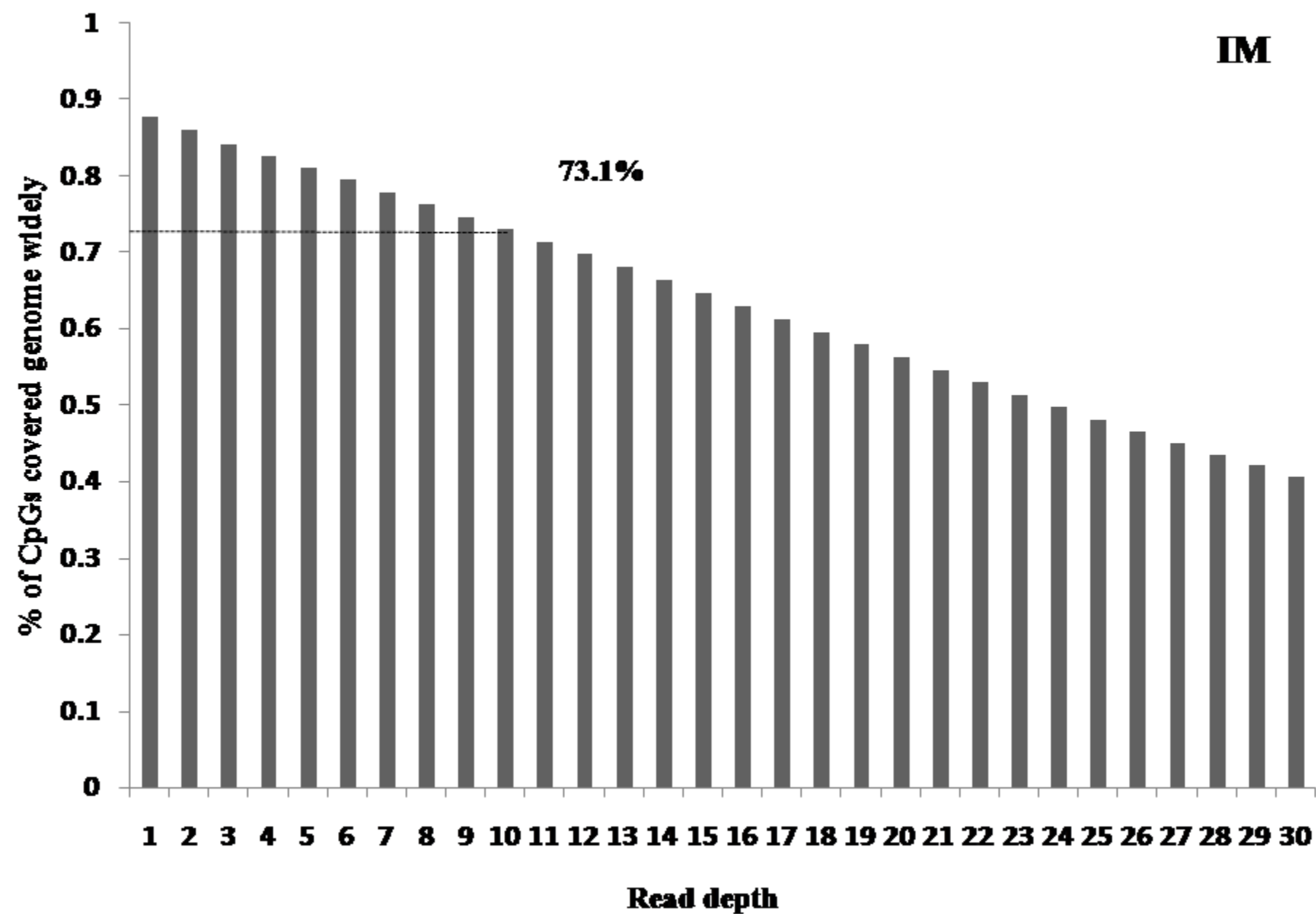

Supplement: S1 File — A: control females (CF). B: control males (CM). C: high temperature-treated females (TF). D: high temperature-induced males (IM). LINE: Long interspersed nuclear elements. SINE: Short interspersed nuclear elements (PDF) [file pone.0158483.s002.pdf]

A

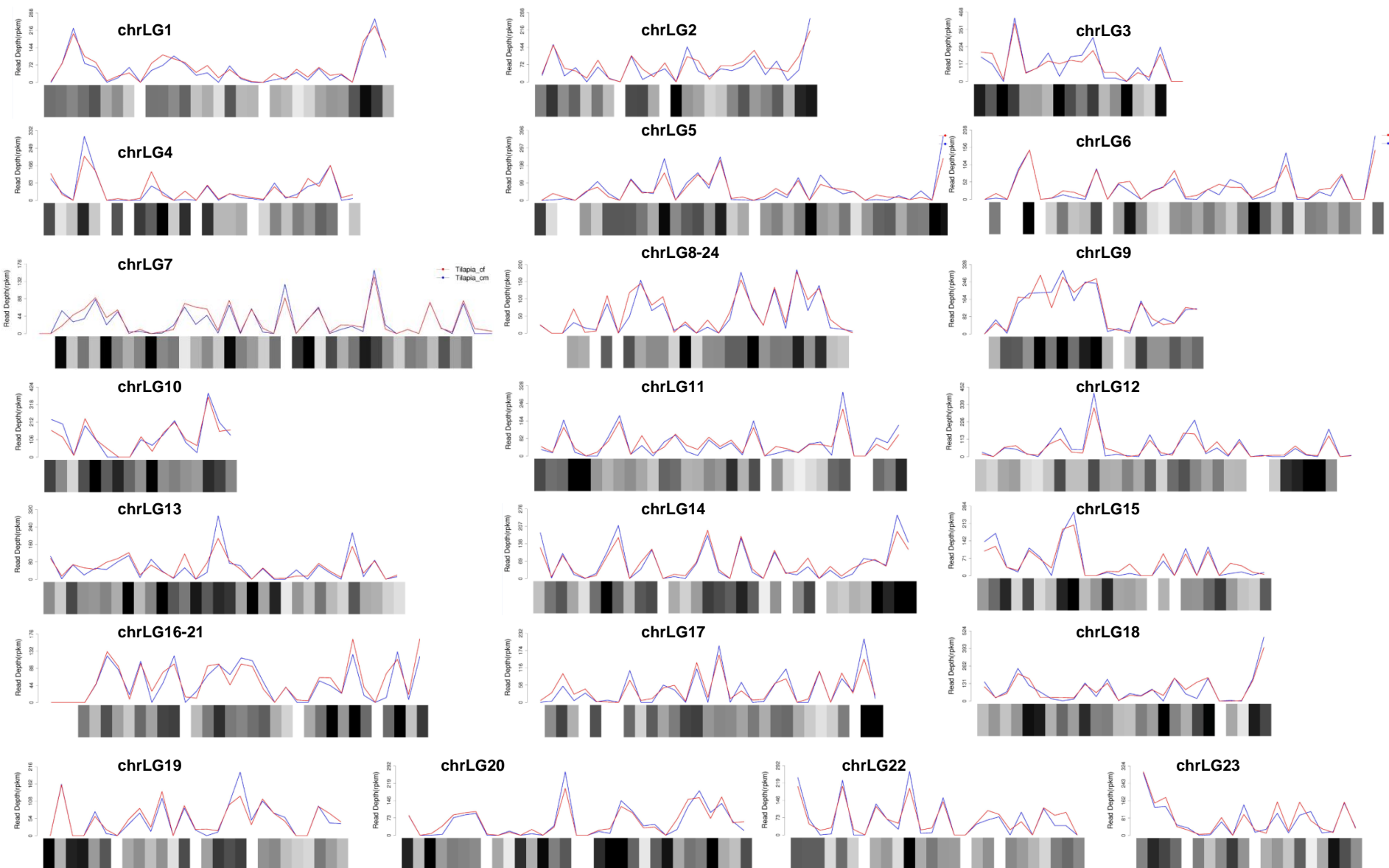

B

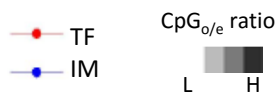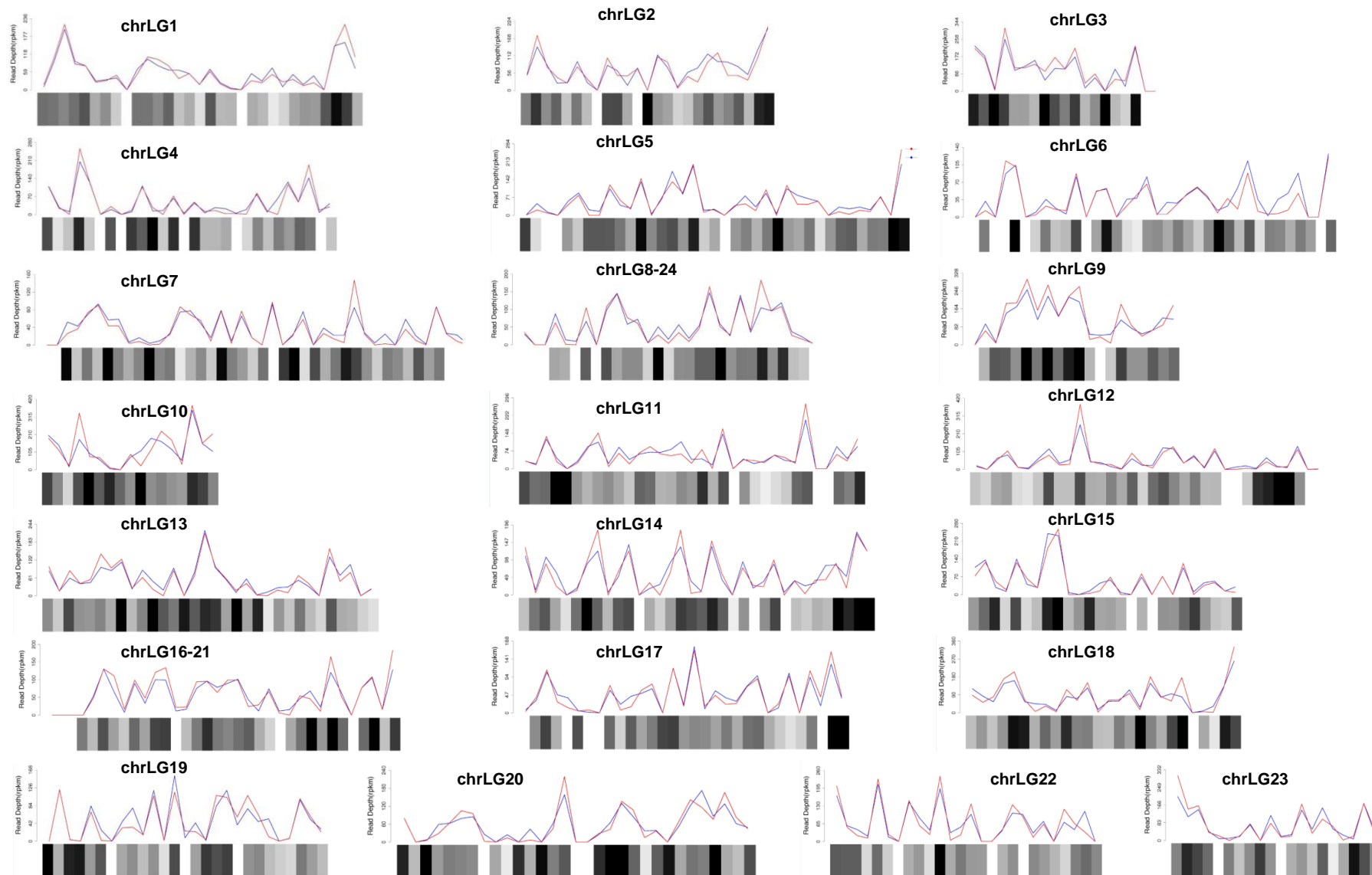

C

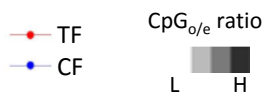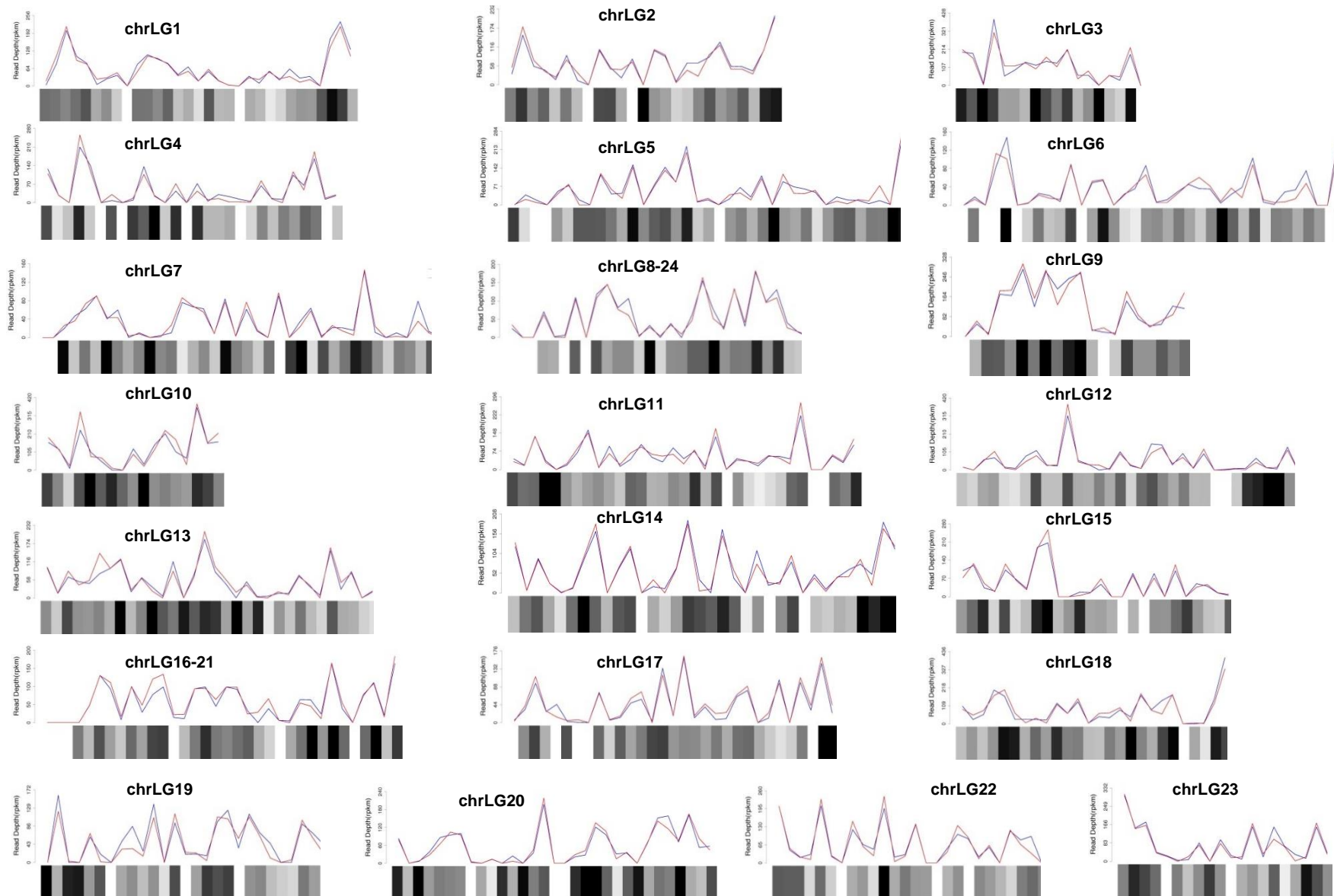

D

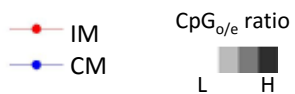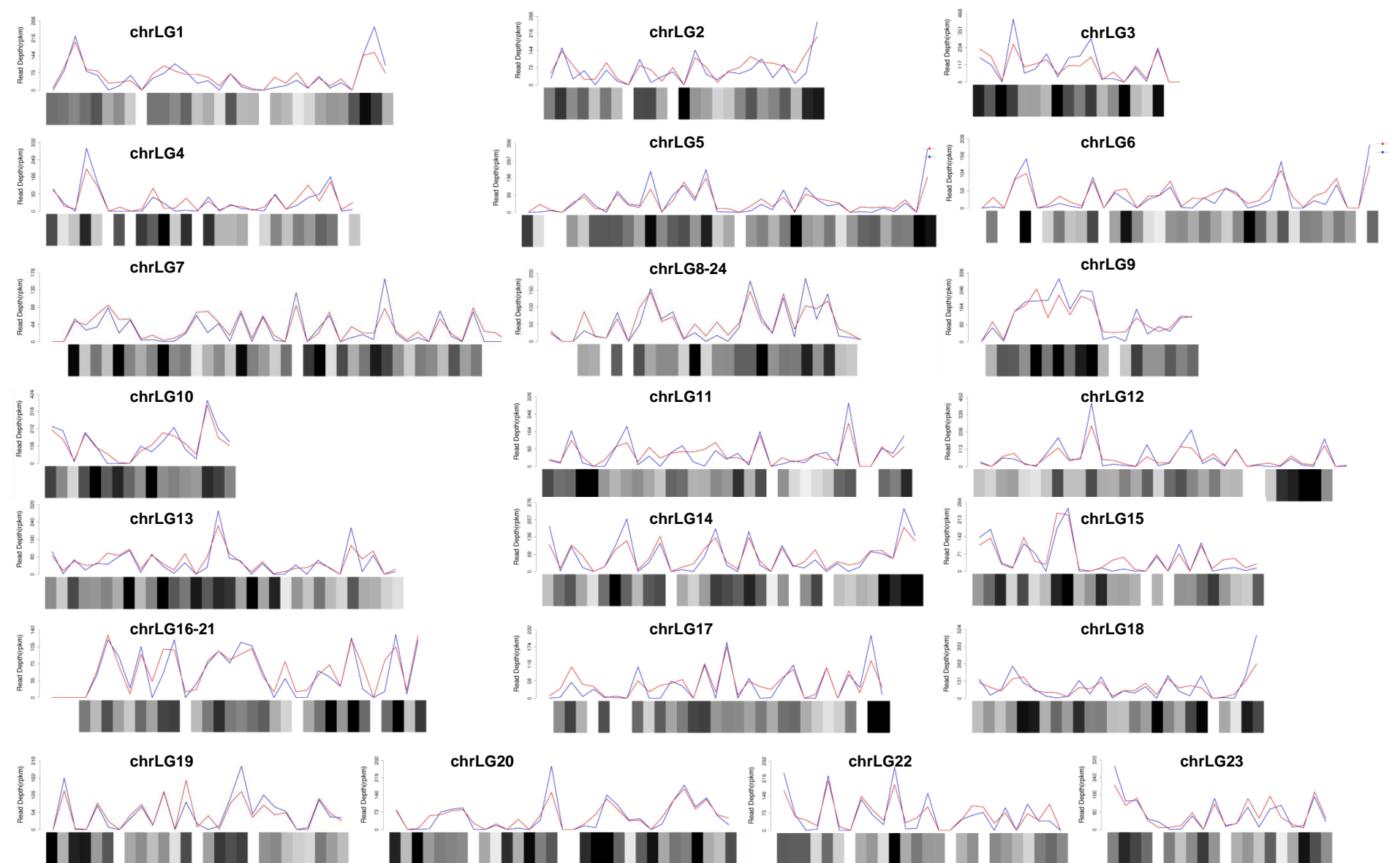

Supplement: S2 File — A: CF/CM; B: TF/IM; C: TF/CF; D: IM/CM.CF: control females. CM: control males. TF: high temperature-treated females. IM: high temperature-induced males. (PDF) [file pone.0158483.s003.pdf]
