# Supplementary material for: Global DNA Methylation Changes in Nile Tilapia Gonads during High Temperature-Induced Masculinization
Source: PLoS One. 2016 Aug 3;11(8):e0158483. doi: 10.1371/journal.pone.0158483 (PMC4972363; doi:10.1371/journal.pone.0158483)
Supplement: S7 Table — (DOCX) [file pone.0158483.s010.docx]

**S9 Table: The primers for BSP.**

| Primer pairs | Sequence (5’→3’) | Amplicon length/bp | Annealing temperature | Purpose |
| --- | --- | --- | --- | --- |
| hsd17b8-F1  hsd17b8-R1  hsd17b8-F2  hsd17b8-R2  gpr54-F1 | TATGAAAGTTTTTTGAAAGATGAAAGT  TAATTAATTTTCCTTATTTACTATATAAAT  GGATATTGTGAATTGGATTTTAGTAT  AAACCTTTAAAAAAACACACATAATT  TTGATTGTTATTATTTTGGTAATATT | 386  386  197  197  325 | 51  51  52  52  47 | For first cycle PCR  For second cycle PCR  For first cycle PCR |
| gpr54-R1 | TTTTCCTAAAAAACTTTTAAAAAC | 325 | 47 |  |
| gpr54-F2 | GTGAATTTTTTGTTTGAGAGAATAAT | 237 | 52 | For second cycle PCR |
| gpr54-F2 | AACTCCCATAAAACCATAAACTATAA | 237 | 52 |  |
