# Supplementary material for: Global DNA Methylation Changes in Nile Tilapia Gonads during High Temperature-Induced Masculinization
Source: PLoS One. 2016 Aug 3;11(8):e0158483. doi: 10.1371/journal.pone.0158483 (PMC4972363; doi:10.1371/journal.pone.0158483)
Supplement: S8 Table — (DOCX) [file pone.0158483.s011.docx]

**S10 Table: The primers for RT-qPCR.**

| Primer pairs | Sequence (5’→3’) | Amplicon length/bp | Annealing temperature | GenBank No. (Gene symbol) |
| --- | --- | --- | --- | --- |
| hsd17b8-F | GCATCACTCAGGACGACTTC | 225 | 60 | NM001279536 |
| hsd17b8-R | TTAGAGGCAGCGTAGTTAGC | 225 | 60 | NM001279536 |
